# Mechanical behavior and constitutive model for loess samples under simulated acid rain conditions

**DOI:** 10.1038/s41598-022-08199-9

**Published:** 2022-03-09

**Authors:** Xia Ye, Cong Chen, Enlong Liu, Baofeng Di, Yanyang Yu

**Affiliations:** 1 Institute of Disaster Management and Reconstruction, Sichuan University, Chengdu, 610041 China; 2 State Key Laboratory of Hydraulics and Mountain River Engineering, College of Water Resource and Hydropower, Sichuan University, Chengdu, 610065 Sichuan China

**Keywords:** Solid Earth sciences, Engineering

## Abstract

Acid rain is mainly composed of sulfuric acid and nitric acid aqueous solutions, which can deteriorate the mechanical properties of soil and thus threaten the safety of soil engineerings. In this paper, the influence of sulfuric acid rain on mechanical properties of loess soil samples was studied. The diluted sulfuric acid solution has respectively pH 5.0, 4.0 and 3.0 to simulate the acid rain condition, and the triaxial compressional tests and scanning electron microscope were carried out to investigate the deteriorated properties and evolution of the microstructure of the saturated loess samples. The results demonstrated that acid rain made the porosity of loess samples larger, and changed the pore distribution and contacts of soil grains, so that the mechanical properties of loess samples varied in some degree. With the decrease of pH value, both the peak value of the deviatoric stress and volumetric contraction of loess samples decreased, which reduced the parameters of shear strength of loess samples. Furthermore, a framework of the chemical–mechanical model for loess under the action of acid rain was established, in which loess was considered as a porous medium material, and the influences of acid rain with different pH values were taken into account in the double-hardening constitutive model, and the model was also verified by the triaxial test results finally.

## Introduction

In recent years, the problem of acid rain has been becoming common, which will not only damage the building structures, but also aggravate soil pollution^1,2^. As we know, acid rain can corrode buildings, roadbed and pavement, dissolve soils on the surface of roadbed and pavement, which can weaken the mechanical features of soil and buildings, and thus may destroy the roadbed and pavement. In the western zone in China, acid rain affects the mechanical features of loess greatly and thus have a potential threat to the safety of buildings and roads there. Therefore, the effect of acid rain on the mechanical properties of loess samples should be studied, which can provide a guidance for protecting the building located in loess areas there.

A large number of experimental studies have found that acid rain has a severe impact on the mechanical features of geological materials. In terms of rocks, the indoor uniaxial, shear and triaxial tests on rock samples corroded in acid rain environment with different pH values were carried out, and found that it significantly reduced the strength of rock samples and caused damage to rock structures, in which the corrosion mechanism of acid rain on rock samples was also preliminarily discussed^3–5^. The influence of acid rain on the stability of slope was also explored, and relevant chemical weathering experiments under the action of acid rain were conducted to analyze the weakening mechanism of acid rain on slope stability^6,7^. The leaching effect of acid rain on calcareous sandy loam was simulated in which acid rain with different pH aggravated the loss of phosphorus in the soil^8,9^. Huang et al.^10^ found that high temperature and acid chemical solution could affect the mechanical characteristics of rock mass through the physical thermophysical tests and Brazilian splitting tests of red sandstone samples. The changes of the mechanical properties of composite fine grained soil under the influence of acids with different pH values were investigated, and found the compressive strength and shear strength parameters of the soil reduced due to the change of internal structure of the soil resulted from acid rain^11^. Some scholars conducted experiments on the influence of acid rain on soil compression characteristics, which led to significant changes in mineral structure with the decrease of pH values^12–18^. Liu et al.^19^ and Wang et al.^20^ used the laboratory permeability test to study the permeability characteristics under the action of acid erosion, found the influence of acid concentration on the permeability coefficient, and analyzed its mechanism from the perspective of microstructure changes. It can be seen that the erosion of acid rain will damage the structure of geological materials and has a vital effect on the mechanical properties of soil. The above-mentioned scholars mainly conduct related experiments on rock and soil by acid rain, but study on the influences of acid rain on the strength of loess samples were few. Therefore, relevant work needs to be carried out further.

In recent years, some scholars have studied the constitutive relationships of geological soils through the theory of porous media^21–26^, among which constitutive models of the chemical–mechanical behavior of soils were also proposed. Gawin et al.^27^ and Martinelli et al.^28^ proposed a thermal fluidization model that took into account the fine and microscopic scale of concrete hardening, and a high-precision hydrothermal chemical model for early self-drying phenomena was also proposed^29,30^. These scholars studied the chemical–mechanical model of concrete materials, few of which, however, are related to soil materials. For geological materials, the following scholars have done the related research. For example, Sherwood^31^ modified the basic thermodynamic parameters of Biot consolidation and the chemical potential related to the pore fluid; Hueckel^32^ and Gajo et al.^33^ considered that clay was contaminated in the stress history and studied the electro-chemical–mechanical constitutive equation under the elastoplastic condition from the perspective of mass concentration; Nova et al.^34^ proposed a strain hardening elastoplastic model considering the internal plastic strain, weathering and chemical effects of bonded geomaterials; Boukpeti et al.^35^ proposed a model to consider the chemical plastic behavior of organic polluted liquids on clay under vertical flow; Lei et al.^36^ formulated a chemical–mechanical model for the deformation characteristics of expansive soil considering the change of chemical composition of pore fluid; Song and Menon^37^ established a non-local chemical-hydrodynamic model for unsaturated soil at the same chemical loading rate. These scholars mainly explored the chemical ion exchange that occurred inside the rock and soil, and the chemical concentration changes caused by ion exchange, which were taken into account in the constitutive model.

Even though the above-mentioned scholars have made a lot of contributions to the simulated acid rain tests and the chemical–mechanical models for geological materials. There are few studies on the strength tests of loess samples and their mechanical model considering the internal chemical reaction of acid rain. This paper mainly conducts the triaxial tests of saturated loess samples under simulated acid rain conditions, and in the chemical–mechanical framework the double hardening model is formulated to consider the chemical reactions under the action of acid rain to the saturated loess samples.

## Influences of simulated acid rain on mechanical properties of loess samples

### Materials and test methods

The material used here is loess samples extracted from Xi’an area of China. The intact loess was crushed, passed through a 2 mm sieve, and then made into triaxial samples. All the samples were compacted in 4 layers according to the dry density of 1.7 g/cm^3^, with the diameter of 39.1 mm and the height of 80 mm. Then, the prepared triaxial samples were put into sulfuric acid solution for vacuum suction saturation and soaked for more than 24 h. The test conditions were sulfuric acid solutions with pH values of 5.0, 4.0 and 3.0, and the control group was clean water (pH 6.9).The samples were placed on a strain-controlled triaxial instrument for consolidated drained (CD) tests. The confining pressures were 50, 100, 200, and 400 kPa, respectively. The degree of saturation of the samples tested was more than 0.95 and the loading rate was 0.08 mm/min. There were 64 samples in 16 groups. By using the geotechnical test specification (SL237–1999) to the analysis of the test data, the data of the above samples were averaged and the laws of different acid rain were obtained. Afterwards, the loess samples with different pH values were sprayed with metal powder on the surface after treatment, and the top inner surface microstructure of the cylindrical sample was also studied by the scanning electron microscope (SEM).

### Stress–strain relationships

**Figure 1:**
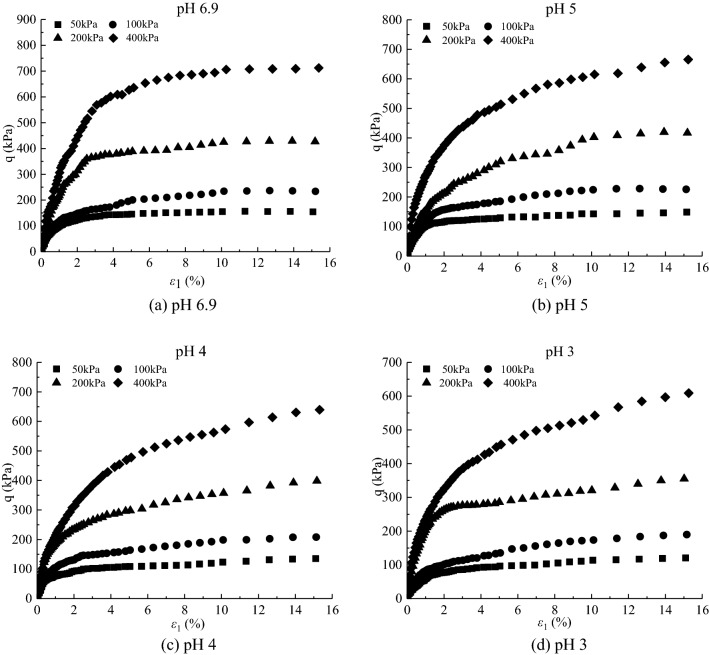
Experimental results of deviatoric stress–axial strain curves of loess samples under different confining pressures.

**Figure 2:**
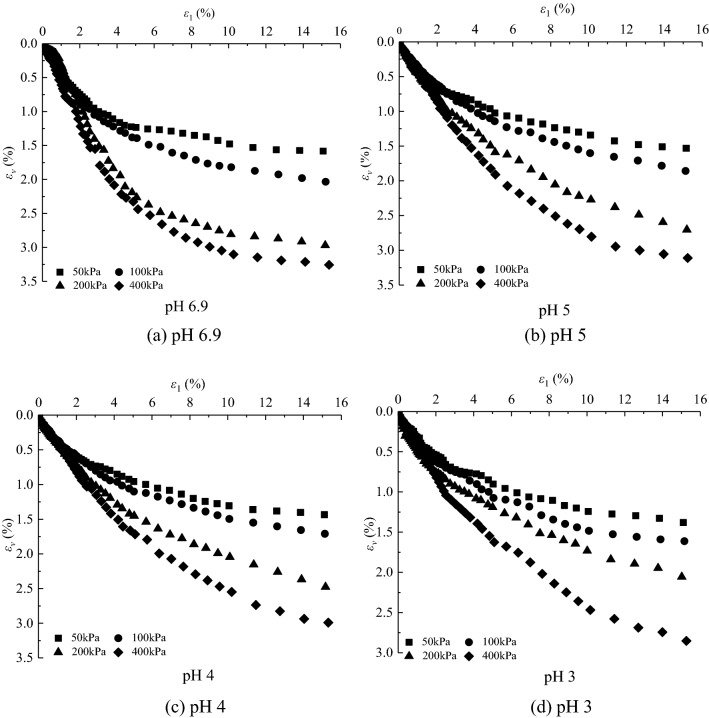
Experimental results of volumetric strain–axial strain curves of loess samples under different confining pressures.

**Figure 3:**
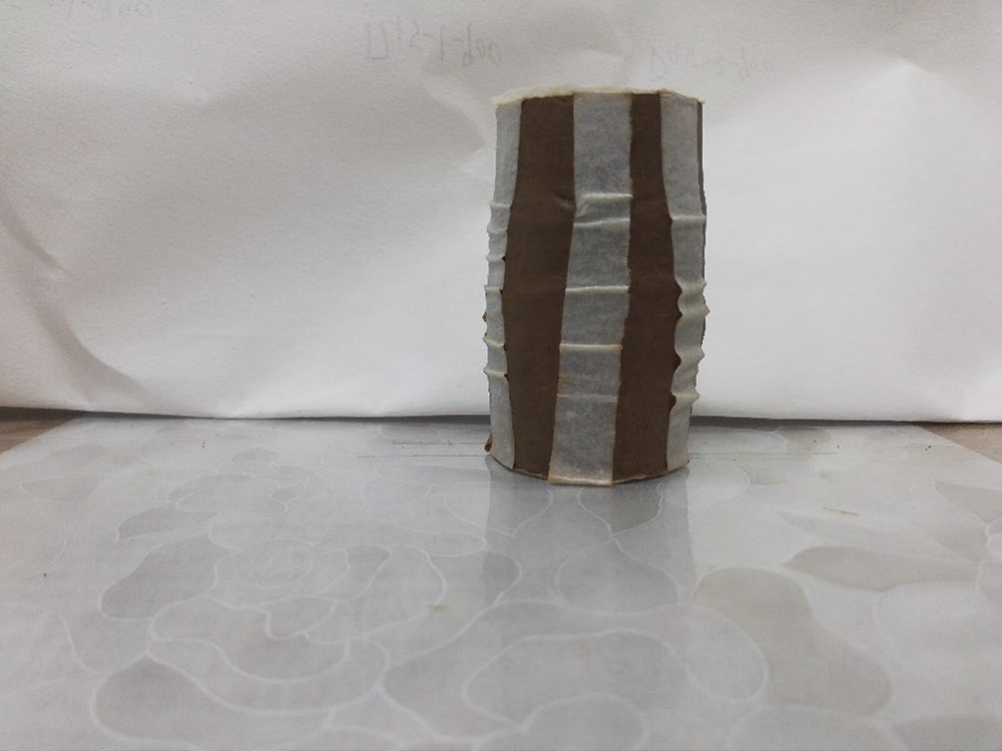
Bulging failure diagram of consolidated drainage sample.

The deviatoric stress $\text{q} = {\upsigma}_{1}-{\upsigma}_{3}$-axial strain ${\upvarepsilon}_{1}$ curves and volumetric strain ${\upvarepsilon}_{\text{v}}$-axial strain ${\upvarepsilon}_{1}$ curves under different confining pressures in the CD tests of saturated loess samples are shown in Figs. 1 and 2, respectively. And sample failure is shown in Fig. 3. Figures 1, 2 and 3 show that: (i) the loess samples present strain*-*hardening behavior under confining pressures ranging from 50 to 400 kPa; and (ii) the loess samples contract under various confining pressures and all the samples fail with bulge. The higher the confining pressures, the higher the peak value of the deviatoric stress and volumetric contraction. During the consolidation process, the bonds between loess particles are heavily damaged under the action of confining pressures. Thus the strength is mainly contributed by the sliding of the soil particles in the shearing process, which leads the loess samples to present strain-hardening behavior and contraction. The higher the confining pressure, the more the bonds damage of loess particles is at the end of consolidation, which caused larger slippage of loess particles in the shear process and thus a larger peak value of deviatoric stress.

**Figure 4:**
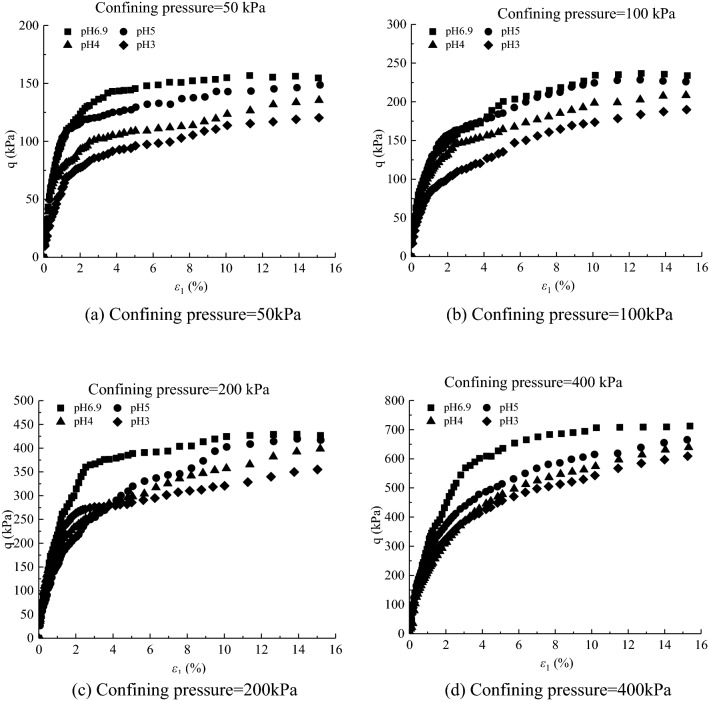
Experimental results of deviatoric stress–axial strain curves of loess samples under different values of pH.

**Figure 5:**
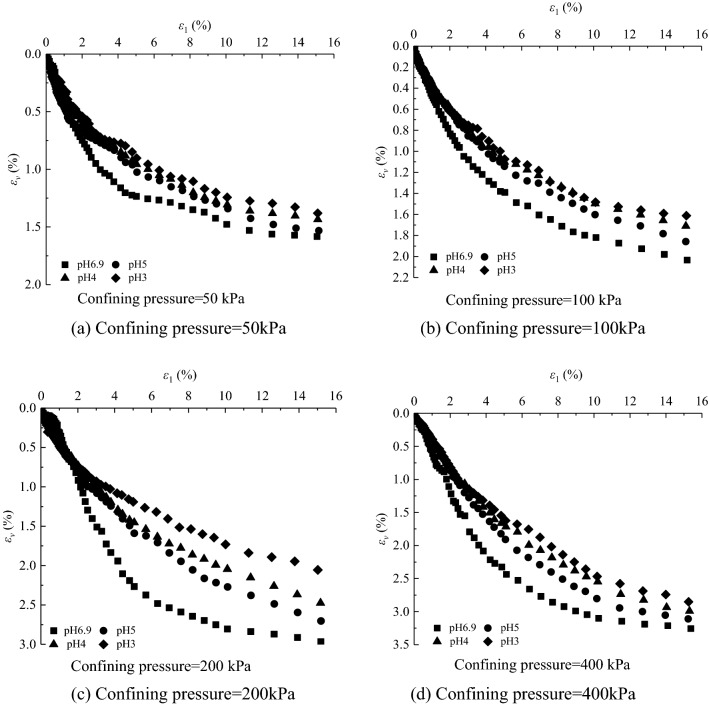
Experimental results of volumetric strain–axial strain curves of loess under different values of pH.

**Figure 6:**
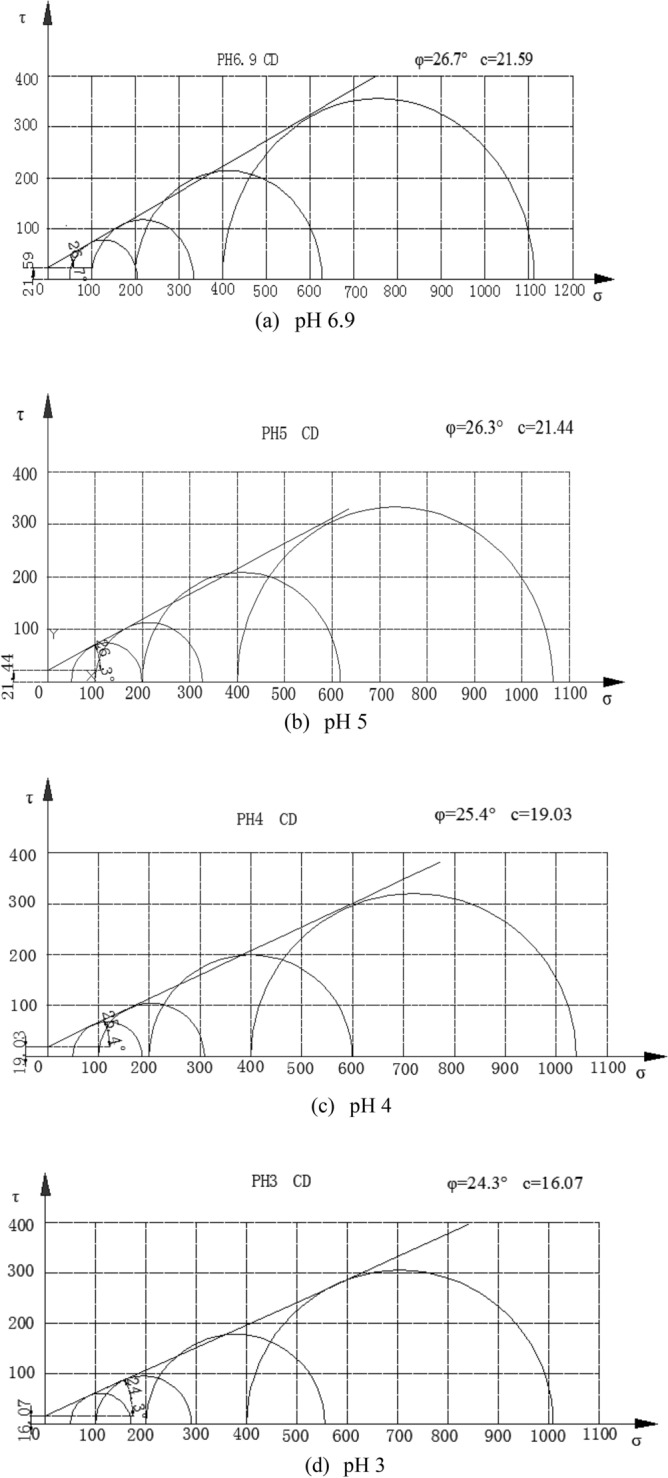
The Mohr stress circle of loess samples under acid rain conditions.

The deviatoric stress ${\text{q}} \, \text{=} \, {\upsigma}_{1}-{\upsigma}_{3}$-axial strain ${\upvarepsilon}_{1}$ curves and volumetric strain ${\upvarepsilon}_{\text{v}}$-axial strain ${\upvarepsilon}_{1}$ curves under different acidic conditions in the CD tests of saturated loess samples are shown in Figs. 4 and 5 respectively. It can be concluded that the mechanical properties of loess samples are significantly affected by the pH value, and (i) under confining pressures ranging from 50 to 400 kPa, the deviatoric stresses of the loess samples under acidic conditions are smaller than those under clear water at a given axial strain; and (ii) the volumetric contraction of the loess samples under acidic conditions is smaller than that under clear water at a given axial strain under various confining pressures. The Mohr stress circle is drawn in Fig. 6, with which the values of internal friction Angle $\varphi$ and cohesion *c* are obtained in Table 3. The expressions of *c-*$\mathit{lg}\text{s}$ and ${\varphi }$-$\mathit{lg}\text{s}$ value are obtained as follows:

1 $$\varphi =2.0007\left(-\mathit{lg}\text{s}\right)+18.002,$$

2 $$c=1.1964\left(-\mathit{lg}\text{s}\right)+14.082,$$

where *s* is the concentration of sulfuric acid in moles per lite, $\text{pH}=-\mathit{lg}s=-{log}_{10}s$. The Eqs. (1) and (2) show that with the decrease of pH value, both c decreases and $\varphi$ decrease. Due to the action of acid rain, the interior of loess sample is eroded, which leads to the decrease of *c* and $\varphi$. Therefore, compared with clear water, the bonds of loess particles under different acidic conditions are weaker, and the loess samples is more likely to be damaged. The lower the pH value, the lower the peak value of the deviatoric stress, and the lower the volumetric contraction.

### SEM images of loess sample under different acidic conditions

**Figure 7:**
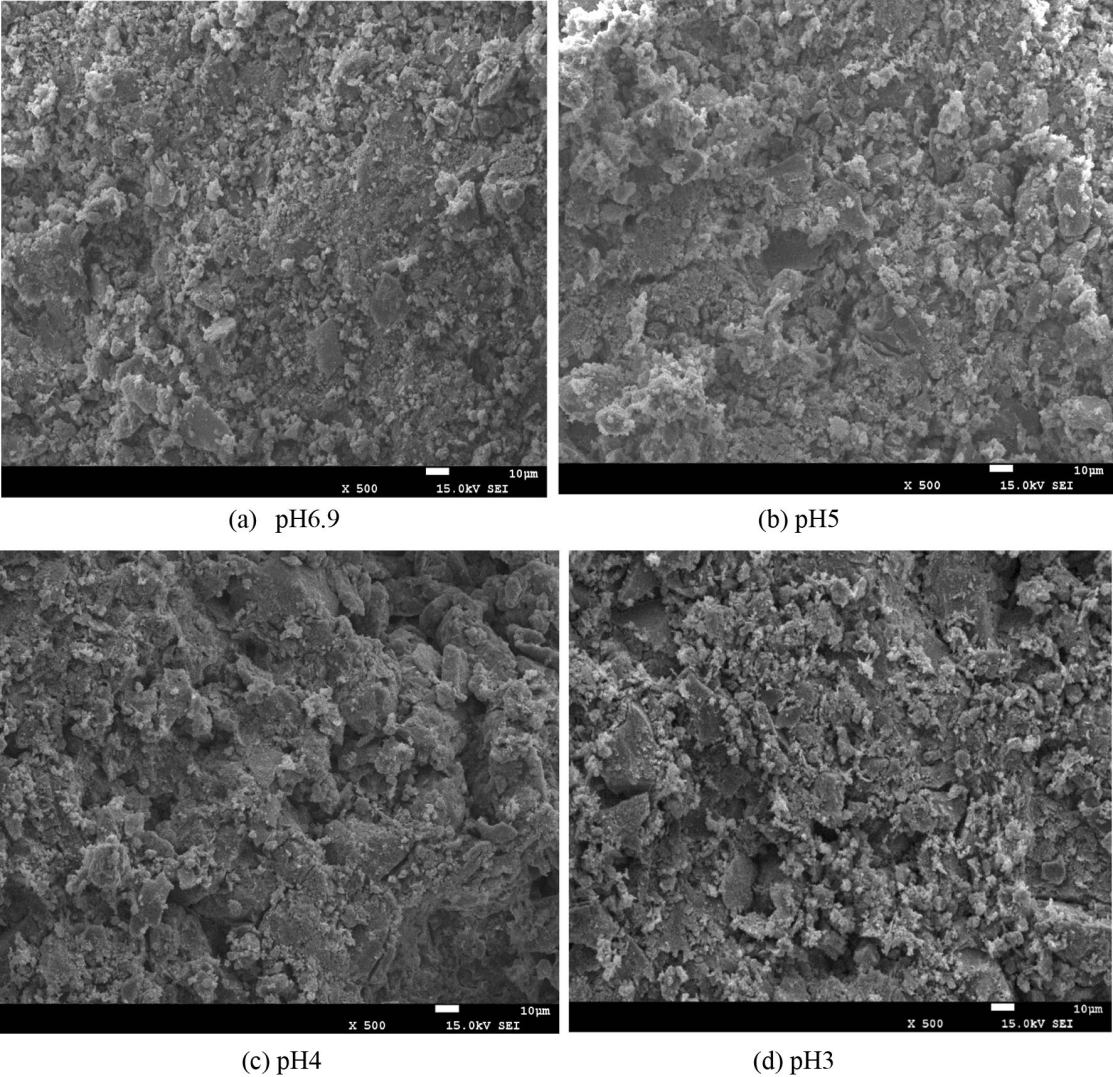
SEM micrographs of loess samples under acid rain conditions.

**Figure 8:**
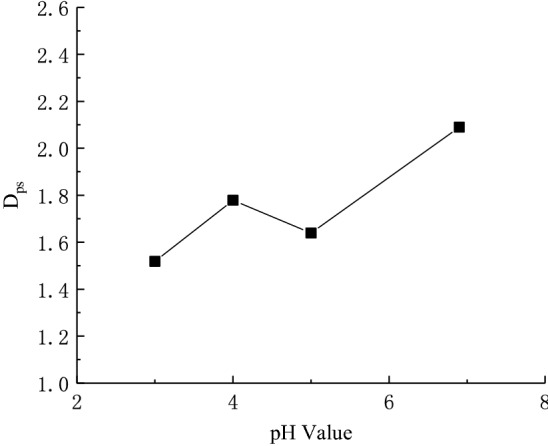
The *D*_*ps*_ of loess samples under acid rain conditions.

Figure 7 shows the microstructure of loess samples after being saturated with clear water and sulfuric acid solutions of different pH values. The microstructure of loess sample has undergone significant changes. It can be seen from Fig. 7a that the soil sample is very dense and has no obvious pores under clear water conditions. It shows that the cementation of immersion in clean water is strong, and a higher deviatoric stress value is required to be destroyed. By comparing Fig. 7a–d, it can be seen that with the decreasing pH value, the sulfuric acid solution corrodes the dense structure into a pore structure, so the pores gradually increase. The area and diameter of soil particles and pores are calculated by SEM images and the number of soil particles on the particle diameter under different PH values are shown in Table 1. And taking the logarithm of the data in Table 1, to get the slope of the linear stability of the system negative expressed as granularity fractal dimension values *D*_*ps*_, The soil particle size distribution fractal under different PH values is shown in the Fig. 8. It can be seen that the smaller the size distribution fractal value of the soil particle is, the more uneven the soil pore is. The number and percentage of soil pores at different pH values are shown in Table 2. It can be seen that the proportion of medium and large pores increases in the presence of acid, so the soil is more prone to instability.

**Table 1:** The number of particles on the particle diameter at different pH values.

pH value	Soil particle diameter (um)								
	> 1	> 1.5	> 2	> 2.5	> 3	> 3.4	> 4	> 10	> 20
6.9	549	310	227	159	124	99	81	9	1
5	403	227	172	119	87	69	56	12	3
4	348	176	114	80	63	50	36	5	2
3	363	194	130	101	83	61	55	17	3

**Table 2:** The number and percentage of soil pores at different pH values.

pH values	The number of micropore (< 1 um)		The number of fine pore (1–4 um)		The number of mesopore (4–16 um)		The number of macrovoid (> 16 um)		The number of total pore	
6.9	240	28.61%	523	62.34%	68	8.10%	8	0.95%	839	100%
5	227	28.13%	495	61.34%	77	9.54%	8	0.99%	807	100%
4	295	33.26%	503	56.71%	80	9.02%	9	1.01%	887	100%
3	296	32.42%	519	56.85%	87	9.53%	11	1.20%	913	100%

## Constitutive model for loess under acid rain conditions

### Constitutive model considering chemical reaction

Complex chemical reactions take place in the process of loess samples immersed in sulfuric acid solution. The main component of sulfuric acid solution is sulfuric acid, and calcium carbonate is involved in the reaction in loess. In this case, the main equations for chemical reaction that occurred can be simplified as follows:

3 $${\text{CaCO}}_{3} + {\text{H}}_{2}{\text{SO}}_{4} = {\text{CaSO}}_{4} + {\text{CO}}_{2} \uparrow + {\text{H}}_{2}\text{O}.$$

For the macroscopic model of chemical reaction with sulfuric acid, it is considered that the loess is a porous material. In the chemical reaction process, the solid part of the material is formed by the precipitation of unreacted loess and the slightly water-soluble CaSO_4_ generated by the reaction. The condition for the reaction to occur is that the aqueous sulfuric acid solution diffuses into the unreacted loess through the precipitate of CaSO_4_ that has been formed. The aqueous sulfuric acid solution is then consumed until the chemical reaction can no longer occur. Therefore, the reaction is controlled by the diffusion of the sulfuric acid solution through the compound layer. In order to be able to take this chemical reaction into account in the constitutive equation, this paper adopts the chemical–mechanical constitutive model to describe the influence of chemical reactions on soil properties from the macroscopic level^38,39^. The loess consisting of skeleton and fluid is a porous medium. The deformation of loess is considered to be caused by the irreversible behavior of the soil skeleton indicated by the plastic strain ${\upvarepsilon}^{\text{p}}$ and the hardening variable $\upchi$. In addition, for the fluid phase saturated porous space of loess, the sulfuric acid solution is reactive and can react with loess. Mass conservation of sulfuric acid solution:

4 $$\frac{{dm}_{sa}}{dt}=-{\nabla }_{X}{M}_{sa}+{m}_{\to sa}^{^\circ },$$

where ${{\text{d}}{\text{m}}}_{\text{sa}}$ is the change in the mass of the sulfuric acid per unit of macroscopic volume; ${\nabla }_{X}{M}_{sa}$ is the external rate of sulfuric acid fluid mass supply; ${\text{m}}_{\to sa}^{^\circ }$ is the rate of mass consumption of the sulfuric acid during the chemical reaction.

Considering the early immersion of soil in acid solution, the dissipation is mainly caused by the irreversible skeleton evolution of loess and the chemical reaction between sulfuric acid solution and calcium carbonate, so the expression is as follows:

5 $${{\Phi }^{\prime}}={\Phi }_{\to sa}+{\Phi }_{s} {=}{\upsigma}\text{:}\frac{d\varepsilon }{dt} {+} p\frac{d\phi }{dt}-\text{S}\frac{dT}{dt} {-A}_{m}^{{\prime}}\frac{d\xi }{dt}-\frac{d\psi }{dt}\ge 0 ,$$

6 $${{\Phi }_{\to sa}=A}_{m}^{{\prime}}\frac{d\xi }{dt}\ge 0 ,$$

where ${\Phi }_{s}$ is the skeleton dissipation; ${\Phi }_{\to sa}$ is the dissipation related to the chemical reaction between sulfuric acid solution and calcium carbonate, ${{A}}_{{m}}{^{\prime}}$ is the affinity of the chemical reaction, which is related to chemical potential difference; $\xi$ is the degree of chemical reaction, which is related to the mass reaction rate and the unit is mol. The diffusion of sulfuric acid solution through the compound layer is the main mechanism to control the reaction between sulfuric acid and calcium carbonate.

Free energy is expressed by temperature, the total strain, plastic strain, $\phi \text{,}$ hardening parameters and the degree of chemical reaction:

7 $$\psi =\psi \left(T, \varepsilon ,{\varepsilon }^{p},\chi ,\phi ,\xi \right).$$

By substituting the free energy into dissipation, we can get:

8 $${\Phi }^{{\prime}}= \left(\sigma -\frac{\partial \psi }{\partial \varepsilon }\right):d\varepsilon -\left(S+\frac{\partial \psi }{\partial T}\right)dT -\left({A}_{m}^{{\prime}}+\frac{\partial \psi }{\partial \xi }\right)d\xi +\left(p-\frac{\partial \psi }{\partial \phi }\right)d\phi +\sigma :d{\varepsilon }^{p}+\zeta d\chi \ge 0.$$

Referring to Coussy^40^, from (8) we can obtain the following:

9 $$\sigma =\frac{\partial \psi }{\partial \varepsilon }=-\frac{\partial \psi }{{\partial \varepsilon }^{p}} ,S=-\frac{\partial \psi }{\partial T}, p=\frac{\partial \psi }{\partial \phi }, \zeta =-\frac{\partial \psi }{\partial \chi } {, A}_{m}^{{\prime}}=-\frac{\partial \psi }{\partial \xi }. $$

Alternatively, use of energy G defined by:

10 $$G={\psi }_{\text{s}}-p\phi .$$

Formula (9) becomes:

11 $$\sigma =\frac{\partial \text{G}}{\partial \varepsilon } ,S=-\frac{\partial G}{\partial T},\phi =-\frac{\partial G}{\partial p}, \zeta =-\frac{\partial G}{\partial \chi } {, A}_{m}^{{\prime}}=-\frac{\partial G}{\partial \xi }.$$

By differentiating the above state equation, a complex state equation can be obtained:

12 $$d\sigma =\frac{{\partial }^{2}G}{\partial {\varepsilon }^{2}}:\left(d\varepsilon -d{\varepsilon }^{p}\right)+\frac{{\partial }^{2}G}{\partial \varepsilon \partial p}\text{d}p+\frac{{\partial }^{2}G}{\partial \varepsilon \partial T}dT+\frac{{\partial }^{2}G}{\partial \varepsilon \partial \xi }d\xi .$$

In the process of reaction between sulfuric acid solution and remolded loess, the temperature change can be ignored, so d*T* = 0。For the isotropic case, the Eq. (12) becomes:

13 $$ d\varepsilon -d{\varepsilon }^{p}=\frac{1}{3G}d{\sigma }_{s}+(\frac{1}{K}d{\sigma }_{m}-{\beta }_{s}d\xi )1,$$

where ${\sigma }_{m}^{{\prime}}=\frac{1}{3}({\sigma }_{1}+2{\sigma }_{3})$, ${\sigma }_{s}={\left(\frac{3}{2}{s}_{ij}{s}_{ji}\right)}^{1/2}={\sigma }_{1}-{\sigma }_{3} ,{\sigma }_{m}={\sigma }_{m}^{{\prime}}-p$. The axial stress, confining pressure, the mean stress and the shear stress under the condition of triaxial compression are denoted by ${\upsigma}_{1}$, ${\upsigma}_{3}$, ${\sigma }_{m}^{{\prime}}$ and ${\upsigma}_{\text{s}}$. The deviatoric tensor of stress are denoted by ${s}_{ij}$.

The standard principle of ideal plasticity is written:

14 $$\sigma \in {C}_{E}\Leftrightarrow F(\sigma ,p,\zeta )\le 0.$$

In the formula, $F(\sigma ,p,\zeta )$ is the loading function. When the loading point remains constant at the boundary of ${\text{C}}_{\text{E}}$ in the elastic domain ($F=dF=0$), the plastic variable ${\upvarepsilon}^{\text{p}}$ and $\upchi$ will evolve. In a standard plastic model, the hardening development often only relates to the plastic hardening variable $\chi (\zeta =\zeta (\chi ))$, but to the loess dipped in acid rain has also been chemical hardening phenomenon. Therefore, the hardening force is expressed by hardening variables and the degree of chemical reaction:

15 $$\zeta =\zeta \left(\chi ,\xi \right).$$

With regard to the development of plastic variables, the hardening law and flow law are written:

16 $$d{\varepsilon }^{p}=d\lambda \frac{\partial g(\sigma ,p,\zeta )}{\partial \sigma },$$

17 $$d\chi =d\lambda \frac{\partial g(\sigma ,p,\zeta )}{\partial \zeta }.$$

In the formula, the plastic multiplier is denoted by ${\text{d}}\uplambda$, and the plastic potential is denoted by $g(\sigma ,p,\zeta )$. Because the plastic multiplier d$\lambda$ is not negative, according to the law of hardening and flow, plastic increment ${\text{d}}{\upvarepsilon}^{\text{p}}$ is represented by thermodynamics $\frac{\partial g(\sigma,\,p,\,\zeta )}{\partial \sigma } {\text{and}}\frac{\partial g(\sigma, \,p, \zeta )}{\partial p}$, and $d\chi$ is represented by thermodynamics $\frac{\partial g(\sigma ,p,\zeta )}{\partial \zeta }$. Furthermore, the elastic domain develops and changes because the hardening force is affected by the degree of chemical reaction, even if there is no plastic evolution in the loess specimen ($d\lambda =0$), the hardening variable $\chi$ remains unchanged in d*t* duration. The degree of chemical reaction $\xi$ is defined according to physical chemistry as:

18 $$\xi =\frac{sV-{n}_{B}(0)}{{v}_{B}},$$

where *s* is the concentration of sulfuric acid solution; ${v}_{B}$ is the measurement coefficient of the chemical reaction, and − 1 is used for this reaction; ${n}_{B}(0)$ is the amount of the substance at the beginning of the chemical reaction. It is considered that the initial volume ${V}_{0}$ during the entire reaction is equal to the volume *V* when the reaction is completed, so ${n}_{B}\left(0\right)={s}_{0}V$. Therefore, the above formula becomes:

19 $$\xi =\frac{(s-{s}_{0})V}{{v}_{B}}.$$

For $\xi$ dimensionless treatment is *Z*:

20 $$Z=\frac{\xi }{{N}_{0}}.$$

${N}_{0}$ is the amount of sulfuric acid before the chemical reaction.

### Simulation of triaxial compression tests

In order to model the mechanical features of loess under simulated acid rain conditions, the double hardening model is adopted^41,42^. For triaxial compression, we have ${\varepsilon }_{s}={\left(\frac{2}{3}{e}_{ij}{e}_{ji}\right)}^{1/2}=\frac{2}{3}({\varepsilon }_{1}-{\varepsilon }_{3})$, ${ {\upvarepsilon}}_{\text{v}}\,{=}\,{\upvarepsilon}_{1} + \text{2} {\upvarepsilon}_{3}$. The deviation tensor of strain is denoted by ${e}_{ij}$; the axial strain, shear strain and radial strain are respectively denoted by ${\varepsilon }_{1}$, ${ \varepsilon }_{s}$ and ${\varepsilon }_{3}$. ${\sigma }_{m}$ is the effective stress, ${\sigma }_{m}={\sigma }_{m}^{{\prime}}-p$. When the loess is under triaxial compression conditions: the current elastic interval is written as:

21 $$F({\sigma }_{m},{\sigma }_{s},\zeta )\le 0.$$

The constitutive equation is written as:

22 $$F({\sigma }_{m},{\sigma }_{s},{\zeta }_{1},{\zeta }_{2})=\frac{{\sigma }_{m}}{1-{\left(\frac{\eta }{{\zeta }_{1}}\right)}^{n}}-{\zeta }_{2}=0,$$

where ${\zeta }_{1}({\varepsilon }_{s}^{p},\xi )$, ${\zeta }_{2}({\varepsilon }_{v}^{p},\xi )$ are expressed as a hardening parameter; n is the parameter related to the over-consolidation ratio; and ${\eta =} \frac{{\upsigma}_{\text{s}}}{{\upsigma}_{\text{m}}}$. The expression of the hardening parameter ${\zeta}_{1}\text{(}{\upvarepsilon}_{\text{s}}^{\text{p}}{,\xi)}$, ${\zeta }_{2}({\varepsilon }_{v}^{p},\xi )$ are written as:

23 $${\zeta }_{1}\left({\varepsilon }_{s}^{p},\xi \right)={\alpha }_{c}\left(\xi \right){\alpha }_{m0}\left[1-{a}_{1}\text{exp}\left(\frac{{\varepsilon }_{s}^{p}}{{a}_{2}}\right)\right],$$

24 $${\zeta }_{2}\left({\varepsilon }_{v}^{p},\xi \right)=\left[1-{a}_{3}\left(\frac{\xi }{{N}_{0}}\right)\right]{\sigma }_{c0}exp\left(\beta {\varepsilon }_{v}^{p}\right),$$

25 $${\alpha }_{c}\left(\xi \right)={\alpha }_{0}+{N}_{s0}\frac{\xi }{{N}_{0}},$$

where the material parameters are ${\alpha }_{m0}$, ${a}_{1}$, ${a}_{2}$ and ${a}_{3}$; and the reference pressure is ${\sigma }_{c0}$.

Using the associated flow rule, the yield function F is consistent with the plastic potential function *g*, namely *F* = *g*. We can get the ${d\varepsilon }_{v}^{p}$ and ${d\varepsilon }_{s}^{p}$:

26 $${d\varepsilon }_{v}^{p}=d\lambda \frac{\partial g}{\partial {\sigma }_{m}},$$

27 $${d\varepsilon }_{s}^{p}=d\lambda \frac{\partial g}{\partial {\sigma }_{s}}.$$

Meeting the consistency condition, the expression of ${\text{H}}$ and $d\lambda$ are written as:

28 $$d\lambda =\frac{1}{H}\left[\frac{\partial F}{\partial {\sigma }_{m}}d{\sigma }_{m}+\frac{\partial F}{\partial {\sigma }_{s}}d{\sigma }_{s}+\left(\frac{\partial F}{\partial {\zeta }_{1}}\frac{\partial {\zeta }_{1}}{\partial \xi }+\frac{\partial F}{\partial {\zeta }_{2}}\frac{\partial {\zeta }_{2}}{\partial \xi }\right)d\xi \right],$$

29 $$H=-\frac{\partial F}{\partial {\zeta }_{1}}\frac{\partial {\zeta }_{1}}{\partial {\chi }_{1}}\frac{d{\chi }_{1}}{d\lambda }-\frac{\partial F}{\partial {\zeta }_{2}}\frac{\partial {\zeta }_{2}}{\partial {\chi }_{2}}\frac{{d\chi }_{2}}{d\lambda }.$$

Putting $d{\chi }_{1}={d\varepsilon }_{s}^{p}=d\lambda \frac{\partial F}{\partial {\sigma }_{s}}$ and ${d\chi }_{2}={d\varepsilon }_{v}^{p}=d\lambda \frac{\partial F}{\partial {\sigma }_{m}}$ into (29), it becomes:

30 $$H=-\frac{\partial F}{\partial {\zeta }_{1}}\frac{\partial {\zeta }_{1}}{\partial {\varepsilon }_{s}^{p}}\frac{\partial F}{\partial {\sigma }_{s}}-\frac{\partial F}{\partial {\zeta }_{2}}\frac{\partial {\zeta }_{2}}{\partial {\varepsilon }_{v}^{p}}\frac{\partial F}{\partial {\sigma }_{m}},$$

where

$$\frac{\partial F}{\partial {\sigma }_{m}}=\frac{1-(1+n){(\frac{\eta }{{\zeta }_{1}})}^{n}}{{(1-{\left(\frac{\eta }{{\zeta }_{1}}\right)}^{n})}^{2}},\frac{\partial F}{\partial {\sigma }_{s}}=\frac{n{(\frac{\eta }{{\zeta }_{1}})}^{n-1}}{{{\zeta }_{1}(1-{\left(\frac{\eta }{{\zeta }_{1}}\right)}^{n})}^{2}},\frac{\partial {\zeta }_{1}}{\partial \xi }={\alpha }_{m0}\left[1-{a}_{1}\mathit{exp}\left(-\frac{{\varepsilon }_{s}^{p}}{{a}_{2}}\right)\right]\frac{{N}_{s0}}{{N}_{0}},$$

$$\frac{\partial {\zeta }_{2}}{\partial \xi }=-{\sigma }_{c0}\mathit{exp}\left(\beta {\varepsilon }_{v}^{p}\right)\frac{{a}_{3}}{{N}_{0}},\frac{\partial F}{\partial {\zeta }_{1}}=\frac{-n{\sigma }_{m}{\eta }^{n}}{{{\zeta }_{1}}^{n+1}{\left[1-{\left(\frac{\eta }{{\zeta }_{1}}\right)}^{n}\right]}^{2}},\frac{\partial F}{\partial {\zeta }_{2}}=-1,\frac{\partial {\zeta }_{2}}{\partial {\varepsilon }_{v}^{p}}=\beta {\zeta }_{2},$$

$$\frac{\partial {\zeta }_{1}}{\partial {\varepsilon }_{s}^{p}}=\frac{1}{{a}_{2}}\left({\zeta }_{1}-{\alpha }_{c}\left(\xi \right){\alpha }_{m0}\right) .$$

Hence, the shear and volumetric strain increments are composed of elasticity and plasticity:

31 $$d{\varepsilon }_{v}=d{\varepsilon }_{v}^{E}+{d\varepsilon }_{v}^{p}={A}_{1}d{\sigma }_{m}+{A}_{2}d{\sigma }_{s}+{A}_{3}d\xi ,$$

32 $$d{\varepsilon }_{s}=d{\varepsilon }_{s}^{E}+{d\varepsilon }_{s}^{p}={B}_{1}d{\sigma }_{m}+{B}_{2}d{\sigma }_{s}+{B}_{3}d\xi ,$$

where

$${A}_{1}=\frac{1}{K}+\frac{1}{H}\frac{\partial F}{\partial {\sigma }_{m}}\frac{\partial F}{\partial {\sigma }_{m}}, {A}_{2}=\frac{1}{H}\frac{\partial F}{\partial {\sigma }_{m}}\frac{\partial F}{\partial {\sigma }_{s}},{A}_{3}=\frac{1}{H}\left(\frac{\partial F}{\partial {\zeta }_{1}}\frac{\partial {\zeta }_{1}}{\partial \xi }+\frac{\partial F}{\partial {\zeta }_{2}}\frac{\partial {\zeta }_{2}}{\partial \xi }\right)\frac{\partial F}{\partial {\sigma }_{m}}-{\beta }_{s},$$

$${B}_{1}=\frac{1}{H}\frac{\partial F}{\partial {\sigma }_{m}}\frac{\partial F}{\partial {\sigma }_{s}}, {B}_{2}=\frac{1}{3G}+\frac{1}{H}\frac{\partial F}{\partial {\sigma }_{s}}\frac{\partial F}{\partial {\sigma }_{s}}, {B}_{3}=\frac{1}{H}\left(\frac{\partial F}{\partial {\zeta }_{1}}\frac{\partial {\zeta }_{1}}{\partial \xi }+\frac{\partial F}{\partial {\zeta }_{2}}\frac{\partial {\zeta }_{2}}{\partial \xi }\right)\frac{\partial F}{\partial {\sigma }_{s}} .$$

### Model validation

The parameters of consolidated drained tests are as follows in Table 3: $E={E}_{0}{\left(\frac{{\upsigma }_{\text{c}0}}{{\text{P}}_{\text{a}}}\right)}^{m}$, ${E}_{0}=1099\left(-\mathit{lg}s\right)+1400.4$, $m=-\,0.0196(-\mathit{lg}s) +0.8246$. ${\sigma }_{c0}$ is the reference pressure when ${\varepsilon }_{v}^{p}=0$. In this paper, the confining pressure of the conventional triaxial consolidated drained tests under the corresponding conditions is 50, 100, 200 and 400 kPa, respectively. Standard atmospheric pressure is represented by ${P}_{a}$. The values of K and G are determined by $K=\frac{E}{3(1-2\nu )}$ and $G=\frac{E}{2(1+\nu )}$, Poisson's ratio $\nu =0.2$. The initial pore ratio is represented by ${e}_{0}$, ${e}_{0}=0.712$. $\lambda$ represents the slope of the isobaric consolidation curve, and $\kappa$ represents the slope of the rebound curve. It is available in the $\nu -lnp$ plane$: \lambda =0.088$, $\kappa =0.008$. $\beta =\frac{1+{e}_{0}}{\lambda -\kappa }=21.4$, *n* = 1.4, ${\alpha }_{m0}$ is a model parameter related to n and effective internal friction Angle $\varphi$. $\varphi =2.0007\left(-\mathit{lg}\text{s}\right)+18.002$. The expression is as follows:

**Table 3:** The parameters under the different pH values.

pH value	Friction angle *φ* (°)	Cohesion *c* (kPa)	E (kPa)	${\alpha }_{m0}$	${a}_{1}$	${a}_{2}$	${\alpha }_{0}$	Confining pressure (kPa)
6.9	26.7	21.59	5489.6117	2.4677	0.8971	− 0.0052	1.1016	50
			8844.5216				1.0060	100
			14,249.7442				0.9187	200
			22,958.3032				0.8389	400
5	26.3	21.44	4156.1525	2.4274	0.9140	− 0.0092	1.1016	50
			6882.0988				1.0060	100
			11,395.944				0.9187	200
			18,870.3412				0.8389	400
4	25.4	19.03	3488.0669	2.3371	0.9224	− 0.0113	1.1016	50
			5859.7037				1.0060	100
			9843.88441				0.9187	200
			16,537.0240				0.8389	400
3	24.3	16.07	2842.4836	2.2271	0.9340	− 0.0134	1.1016	50
			4844.51424				1.0060	100
			8256.6241				0.9187	200
			14,071.9662				0.8389	400

33 $${\alpha }_{m0}=1.25\sqrt[n]{1+n}\frac{6\sin\varphi }{3-\sin\varphi }.$$

**Figure 9:**
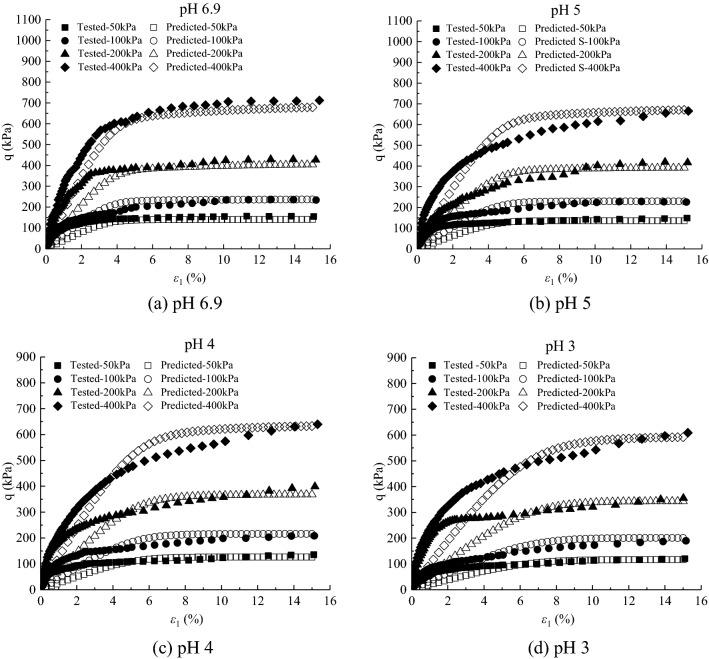
Deviatoric stress–axial strain for tests and simulations.

**Figure 10:**
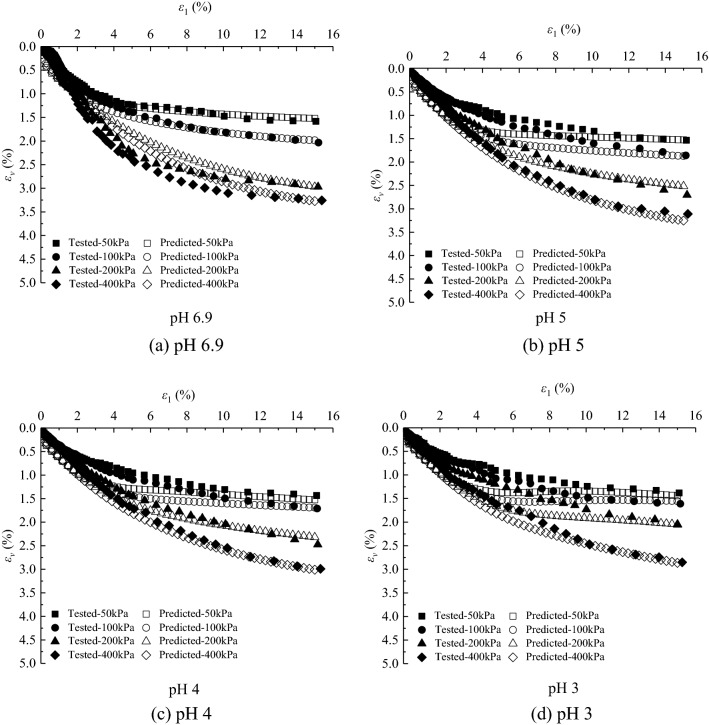
Volumetric strain–axial strain for tests and simulations.

According to the value of the effective internal friction angle under different acidic conditions, the different values of ${\alpha }_{m0}$ can be obtained. ${N}_{s0}=0.0002$, ${a}_{3}=0.001$, ${\beta }_{s}=-0.001$, ${\alpha }_{0}=1.0043{\left(\frac{{\upsigma }_{\text{c}0}}{{\text{P}}_{\text{a}}}\right)}^{-0.131}$, ${a}_{1}=-\,0.0093\left(-\mathit{lg}s\right)+0.9669$, ${a}_{2}=0.0021\left(-\mathit{lg}s\right)-0.0197$. As can be seen from Figs. 9 and 10. The results show that the deviatoric stress–axial strain curves and volumetric strain–axial strain curves of saturated loess can be simulated by a double-hardening model considering the influence of concentration of acid rain. The simulated results of the model under all the confining pressures present strain*-*hardening behavior and contract, which is consistent with the tested results of loess and the peak values are close to the tested results. For the simulated results, the higher the confining pressures, the higher the peak of the deviatoric stress and volumetric contract. The simulated results at different pH values can be the same as the tested results of saturated loess. With the decrease of pH values, the peak of the simulated deviatoric stress and volumetric contract decrease. Although the simulated values are slightly different from the experimental results, the double hardening model can better reflect the deformation behavior of saturated loess.

### Parameter sensitivity analysis

**Figure 11:**
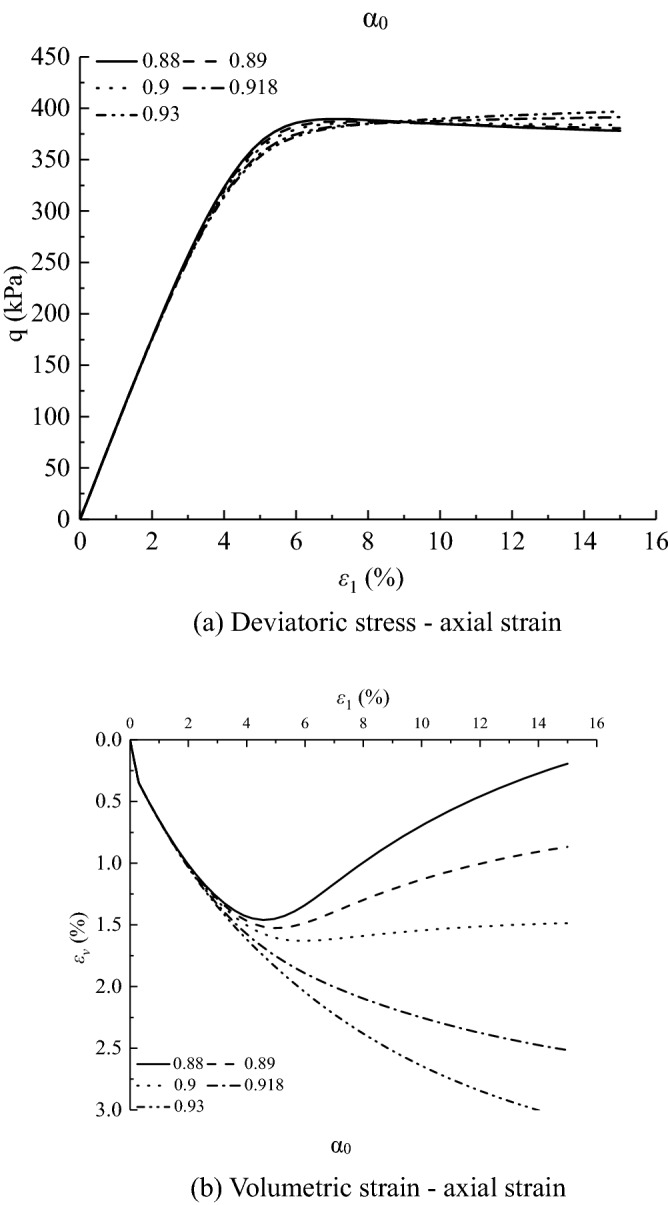
Sensitivity analysis of simulation results with varying ${\alpha }_{0}$.

**Figure 12:**
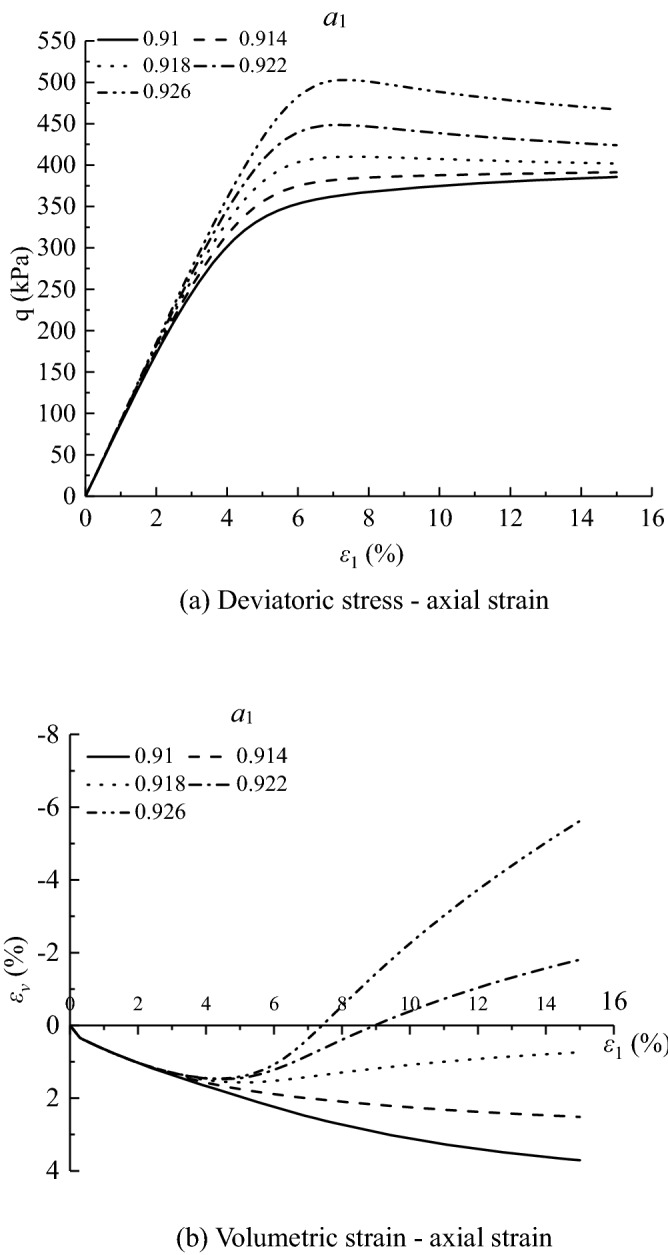
Sensitivity analysis of simulation results with varying ${a}_{1}$.

**Figure 13:**
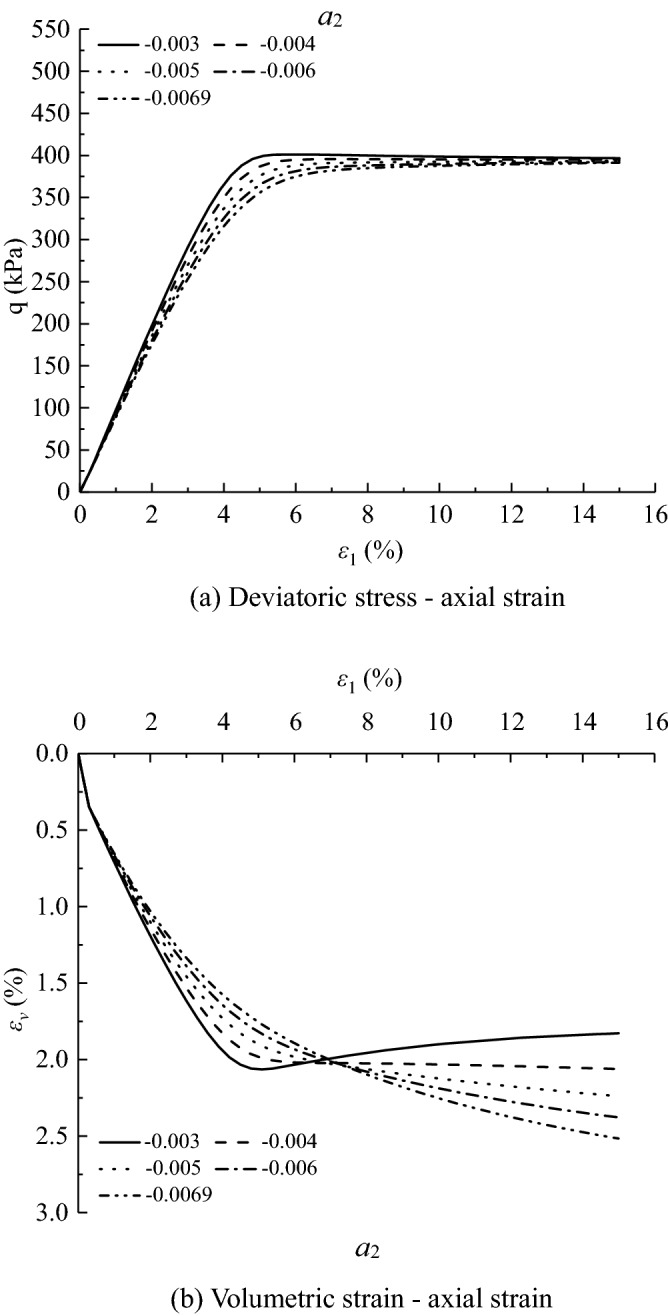
Sensitivity analysis of simulation results with varying ${a}_{2}$.

**Figure 14:**
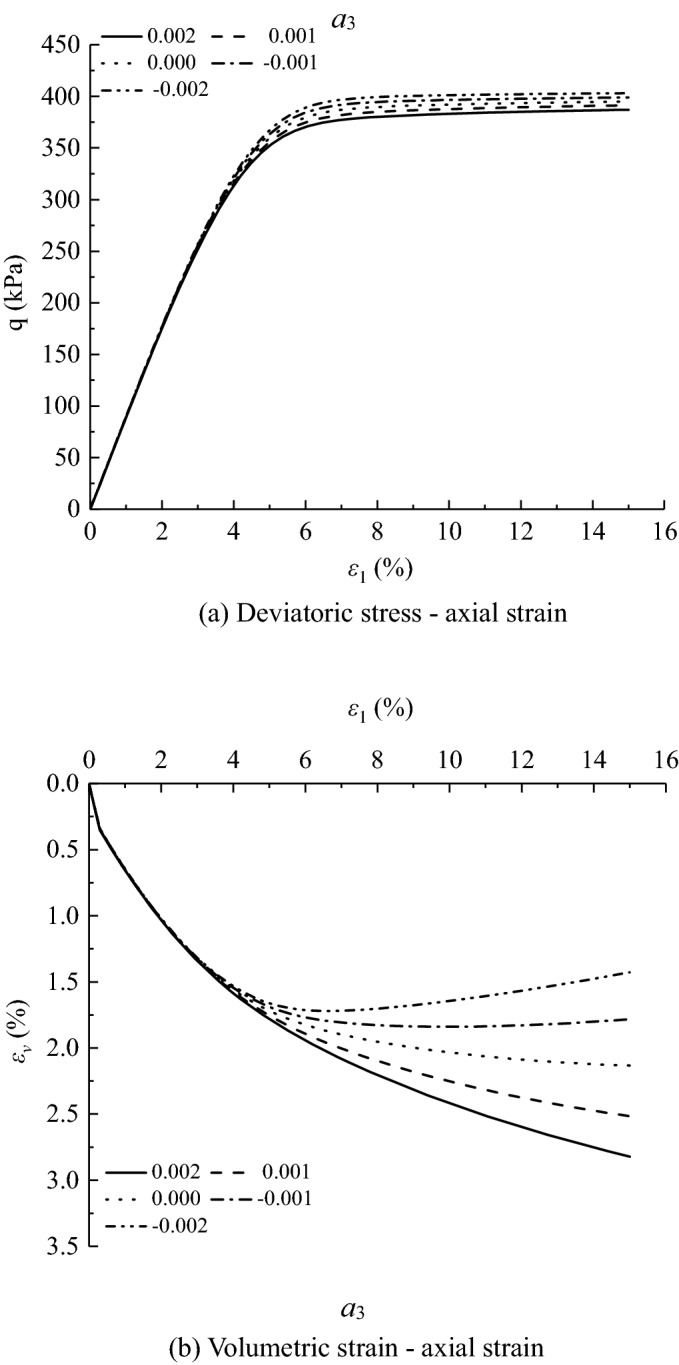
Sensitivity analysis of simulation results with varying ${a}_{3}$.

**Figure 15:**
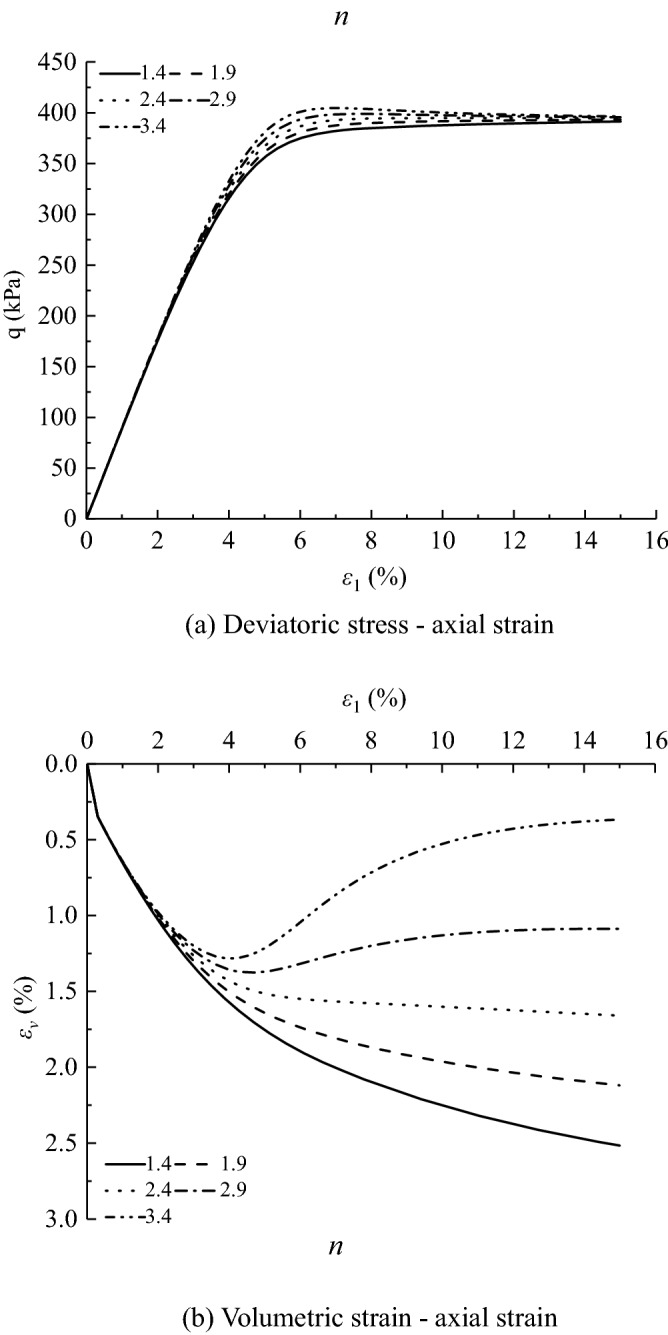
Sensitivity analysis of simulation results with varying n.

**Figure 16:**
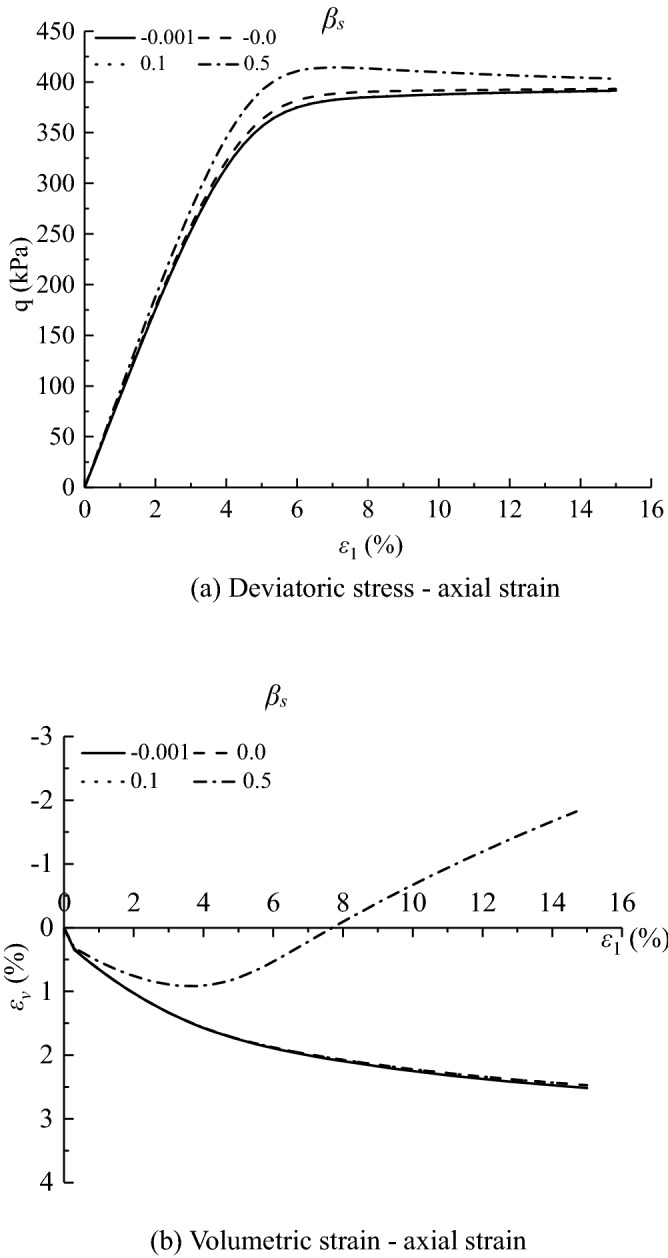
Sensitivity analysis of simulation results with varying ${\beta }_{s}$.

**Figure 17:**
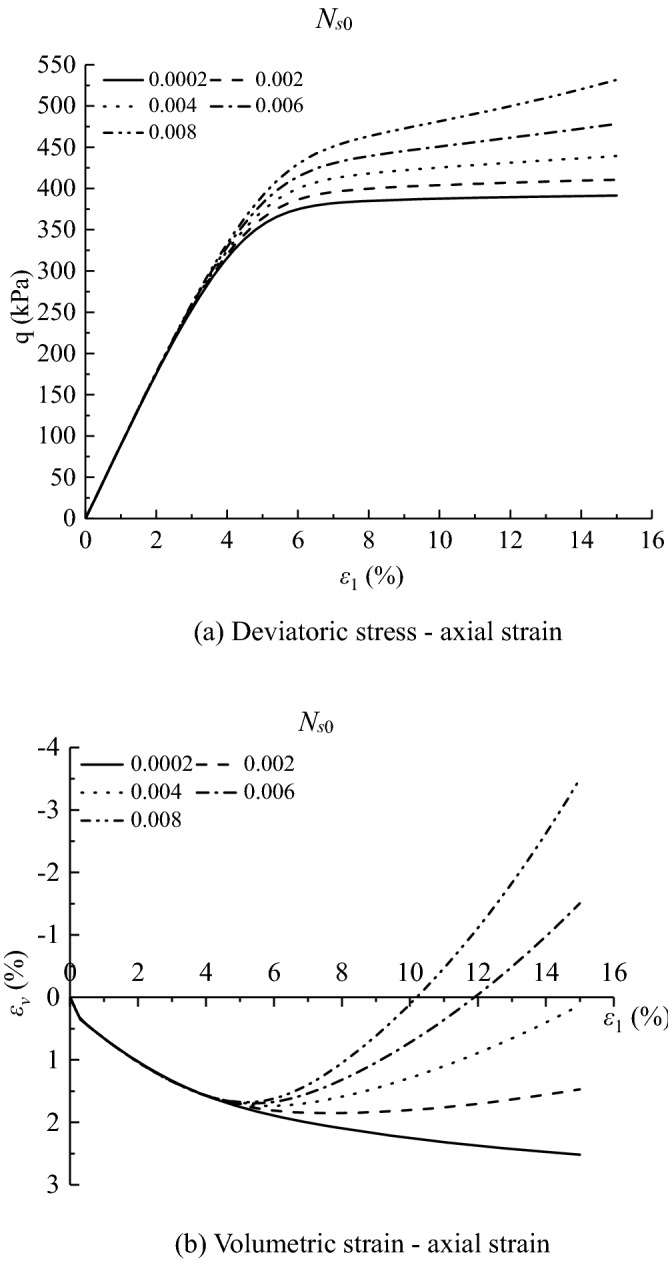
Sensitivity analysis of simulation results with varying ${N}_{s0}$.

Figures 11, 12, 13, 14, 15, 16 and 17 show the calculated results when the model parameters change when the acidic condition is pH 5 and the confining pressure is 200 kPa, including ${a}_{1}$, ${a}_{2}$, ${a}_{3}$, ${\alpha }_{0}$, ${\beta }_{s}$, *n* and ${N}_{s0}$. The selection of model parameters is based on the parameters obtained during the verification of the above model, and the value of the parameters is changed to make the stress–strain relationship curve and the volumetric curve change. The model calculated results can reflect the main mechanical behavior of loess. From the deviatoric stress–axial strain curves, it shows that with the decrease of ${N}_{s0}$ and ${a}_{3}$, the loess samples present strain hardening behavior; With the decrease of ${a}_{1}$, ${\beta }_{s}$ and *n*, the loess samples behave strain softening slightly to strain hardening, and the deviatoric stress peak value decreases; with the reduction of ${a}_{2}$ and ${\alpha }_{0}$, the loess samples behave strain hardening slightly to strain softening. For volumetric strain change, with the decrease of ${a}_{1}$, ${a}_{2}$, ${\beta }_{s}$ and *n*, the loess samples contract more heavily. With the decrease of ${\alpha }_{0}$, ${a}_{3}$, and ${N}_{s0}$, the loess samples dilate more heavily.

## Conclusions

1. By performing the consolidated drained experiments of acid rain on loess samples, it is found that with the increase of acid rain concentration, the peak of the deviatoric stress decreases, and the sample contracts less. The value of *c* and ${\varphi }$ both decrease as the pH decreases. The reason for the reduction of strength is that the cementation failure is caused by acid rain erosion on loess samples through SEM analysis of the microstructure.
2. A thermodynamic framework for the chemical and mechanical coupling of loess under acid rain conditions is proposed. The chemical reaction degree of sulfuric acid and calcium carbonate is taken into account in the framework and the elastoplastic constitutive relationship is established.
3. Introducing the influence of different pH values on the parameters of the double hardening model, the mechanics and deformation behavior of the loess samples under different acid rain conditions can be well simulated by the proposed model.

### Consent to participate

Since this study did not recruit any human subjects, this section does not apply.

### Consent to publish

Since this study is not attempting to re-publish/publish any third party or author’s previously published material, this section does not apply.

### Ethical approval

Since this study did not recruit any human and/or animal subjects, this section does not apply.

## Data Availability

The datasets used and/or analyzed during the current study are available from the corresponding author on reasonable request.

## Code availability

Since this study did not use programming code, this section does not apply.
